# High‐Detectivity MOCVD‐Epitaxial h‐BN‐Based Vacuum UV Photodetectors With Pt Nanoparticle‐Engineered Localized Surface Plasmon Resonance Enhancement

**DOI:** 10.1002/nap2.70070

**Published:** 2026-04-14

**Authors:** Zhiwei Gao, Yufan Wei, Weijie Liu, Yongming Zhao, Zhengang Liang, Yuhui Zeng, Zhencheng Li, Yang Peng, Feng Wu, Hao‐Chung Kuo, Jiangnan Dai

**Affiliations:** ^1^ Wuhan National Laboratory for Optoelectronics Huazhong University of Science and Technology Wuhan China; ^2^ School of Aerospace Engineering Huazhong University of Science and Technology Wuhan China; ^3^ Department of Photonics Institute of Electro‐Optical Engineering National Yang Ming Chiao Tung University Hsinchu Taiwan

**Keywords:** boron nitride, localized surface plasmon resonance, MOCVD, ultrawide‐bandgap semiconductors, VUV photodetector

## Abstract

This study addresses fabrication challenges in hexagonal boron nitride (h‐BN)‐based vacuum ultraviolet photodetectors (VUV PDs) by implementing platinum nanoparticle (Pt NP)‐induced localized surface plasmon resonance (LSPR) effects to enhance photoresponse characteristics. Size‐controlled Pt NPs (32 nm, 50 nm, 85 nm) are synthesized on metalorganic chemical vapor deposition (MOCVD)‐derived h‐BN surfaces through a cost‐effective dewetting process, with systematic evaluation under 185 nm VUV irradiation. The 32‐nm Pt NP‐enhanced device demonstrates optimal performance, achieving 1.02 mA/W responsivity (143% improvement) and 2.95 × 10^11^ Jones specific detectivity (117% enhancement) at 20 V bias. Numerical simulations confirm that LSPR mechanisms intensify localized electric fields and photon absorption efficiency. This work presents the first LSPR‐enhanced h‐BN VUV photodetector utilizing Pt nanoparticles (NPs). The proposed design effectively addresses responsivity limitations in two‐dimensional material‐based detectors. Furthermore, it creates new opportunities for advancing extreme‐environment sensing and imaging technologies. The methodology maintains compatibility with scalable MOCVD fabrication processes, with performance improvements directly correlating to precise NP size optimization, suggesting plasmonic engineering as a critical pathway for next‐generation optoelectronic devices.

## Graphical abstract

MOCVD‐epitaxial h‐BN integrated with size‐controlled Pt nanoparticles achieves enhanced vacuum ultraviolet photodetection via localized surface plasmon resonance. The 32‐nm Pt NP‐optimized device exhibits 1.02 mA/W responsivity and 2.95 × 10^11^ Jones detectivity—representing 143% and 117% improvements. This first LSPR‐engineered h‐BN VUV photodetector establishes plasmonic engineering as a viable strategy for next‐generation two‐dimensional optoelectronics.

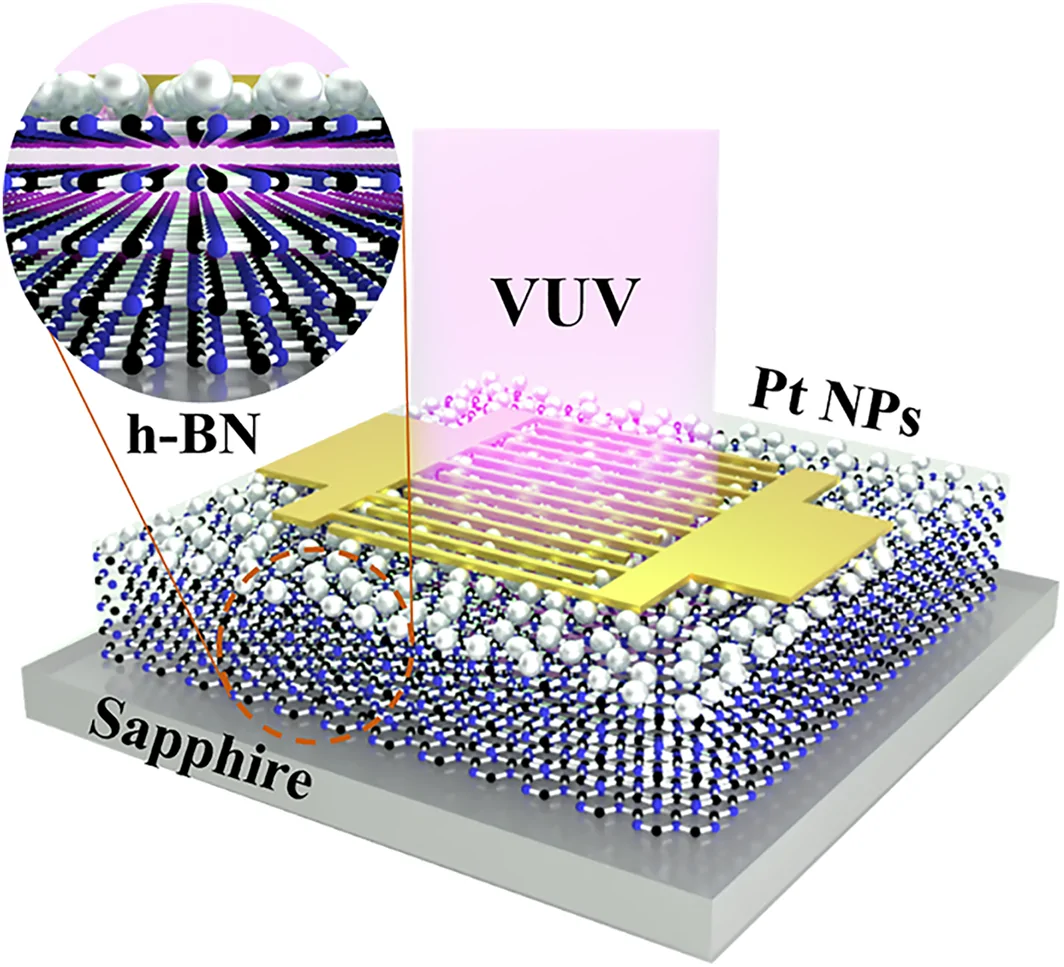

## 1 Introduction

Photodetectors operating in the vacuum ultraviolet (VUV) spectrum (10–200 nm wavelength) play significant roles across diverse disciplines such as optics, materials science, information science, biomedicine, environmental science, and space technology, including solar storm monitoring, deep‐space communications, and analysis of planetary atmospheric constituents [1, 2, 3, 4]. In contrast to conventional ultraviolet chromatographic systems, wide‐bandgap semiconductor (WBGS)‐based VUV photodetectors exhibit more extensive application potential owing to their small footprint, low operating voltages, and excellent spectral selectivity [2, 5, 6]. Present investigations of WBGS materials for VUV photodetection are predominantly centered on GaN, diamond, and Ga_2_O_3_ [7, 8, 9, 10]. Nevertheless, the constrained bandgaps of these materials hinder the realization of selective response to VUV radiation. AlN and h‐BN have garnered widespread interest due to their outstanding optoelectronic characteristics. AlN has been thoroughly studied for this purpose, as evidenced in prior studies [1, 11, 12, 13, 14, 15]. In contrast, BN demonstrates a significantly larger bandgap (∼5.97 eV) that is particularly advantageous for VUV detection. Its band‐edge absorption coefficient reaches 7 × 10^5^ cm^−1^, tripling that of AlN, while maintaining robust thermal stability and radiation tolerance. These combined properties position BN as a promising candidate material for constructing high‐performance VUV PDs. Yet, studies concerning BN thin‐film‐based photodetectors are still comparatively scarce [16, 17, 18]. Mechanically exfoliated h‐BN monolayers and few‐layer systems exhibit exceptional crystallinity and remarkable VUV detection performance. However, achieving precise thickness control and dimensional uniformity during fabrication remains a critical challenge [6, 19]. These technical limitations significantly impede their practical implementation in advanced 2D optoelectronic devices. Although scalable techniques including reactive magnetron sputtering, low‐pressure chemical vapor deposition (LPCVD), and metal‐organic chemical vapor deposition (MOCVD) successfully generate large‐area h‐BN films with controlled stacking architecture, critical material limitations persist. The deposited films frequently contain disordered phases—combining amorphous, turbostratic, and polycrystalline domains—that fundamentally compromise detector responsivity. Furthermore, these structural imperfections continue to hinder practical VUV detection performance, necessitating substantial material engineering improvements [20, 21]. Consequently, innovative processing technologies must be developed to improve the performance of photodetectors based on bottom‐up synthesized h‐BN thin films. Recent advances have revealed that metallic nanoparticle (NP)‐activated localized surface plasmon resonance (LSPR) phenomena exhibit remarkable capabilities for boosting the optoelectronic characteristics of h‐BN‐integrated deep ultraviolet photodetectors (DUV PDs). The LSPR phenomenon enhances optical absorption, leading to increased generation of photogenerated charge carriers that ultimately boost the photocurrent. Zhu and colleagues demonstrated that incorporating Au NPs into a 200‐nm‐thick h‐BN deep UV PD enhanced the responsivity by approximately 7‐fold at a high bias voltage of 35 V, with this enhancement being exclusively observed under positive bias conditions [22]. Kaushik and collaborators [23] employed the LSPR properties of aluminum nanoparticles in the DUV spectral region to construct a boron nitride‐based DUV photodetector, realizing a 5.5‐fold photocurrent enhancement at the wavelength of 205 nm. The device demonstrated approximately 60% enhancement in responsivity at a low bias of 1 V while preserving outstanding UV–visible rejection performance. Nevertheless, the influence of LSPR on the performance of VUV PDs fabricated from h‐BN thin films has not been comprehensively investigated in existing studies.

This work demonstrates the application of LSPR from Pt NPs for substantially improving the performance of VUV PDs fabricated from MOCVD‐derived h‐BN nanosheets. The Pt NPs were synthesized through a cost‐effective dewetting approach. A systematic investigation was conducted on the effect of evaporation‐deposited Pt film thickness on the resulting nanoparticle size, followed by an in‐depth examination of the enhancement mechanisms governing the improved VUV photodetection capabilities in h‐BN films through Pt NP integration. Table 1 compares key performance metrics of VUV PDs using h‐BN and other materials. Our device demonstrates advantages through ultralow dark current, high detectivity, fast response speed, and scalable fabrication feasibility.

**TABLE 1:** Performance comparison of VUV PDs based on h‐BN and other materials.

	*λ*[nm]	*I*_d_[pA]	R[mA W^−1^]	D*[Jones]	*t*_r_/*t*_d_[ms]	Refs.
Amorphous BN	185	0.04@10 V	0.0048@10 V	≈ 2 × 10^9^	5340/1830	[19]
h‐BN thick film	185	≈ 200@90 V	≈ 0.127@90 V	≈ 5.39 × 10^9^	71/65	[21]
h‐BN paper	185	—	≈ 0.05@10 V	8.05 × 10^10^	267/393	[24]
Pt NPs@h‐BN	**185**	**0.10@20 V**	**1.02@20 V**	**2.95 × 10^11^**	**214.15/21.45**	**This work**
2D MgO	150	0.03@6 V	1860@6 V	1.8 × 10^10^	1000/21000	[5]
(AlGa)_2_O_3_/Ga_2_O_3_	198	16@‐10 V	86@‐10 V	1.6 × 10^11^	800/200	[25]
AlN	185	0.05@10 V	6.44@10 V	1.29 × 10^10^	1200/3600	[26]

## 2 Results and Discussion

Figure 1 presents the characterization results of h‐BN films fabricated by MOCVD. Figure 1a displays a cross‐sectional transmission electron microscope (TEM) micrograph of the h‐BN film, in which the highly crystalline h‐BN layer is distinctly visible as the brightest region, exhibiting a homogeneous thickness of 41.26 nm. The high‐resolution transmission electron microscopy (HRTEM) image in Region A clearly reveals the interlayer h‐BN structure, exhibiting atomic‐scale ordering and quasi‐single‐crystalline features. Figure 1b presents the AFM surface morphology of h‐BN, showing uniform ridged protrusions formed by thermal mismatch‐induced wrinkling during cooling. Excessive nitridation arising from prolonged ammonia introduction leads to the formation of white particulates, where suppressed boron adatom mobility facilitates three‐dimensional island growth. Furthermore, Figure 1c shows the in‐plane TEM image of ultrasonically exfoliated h‐BN, where the HRTEM image in Region B reveals in‐plane atomic ordering, confirming superior crystallinity in both interlayer and in‐plane directions for MOCVD‐grown h‐BN. The Raman‐active E_2g_ phonon modes were identified at 1367.8 cm^−1^ for h‐BN and at 417.2, 577.8, and 753.5 cm^−1^ for the sapphire substrate, as depicted in Figure 1d. As evidenced in Figure 1e, the h‐BN film exhibits a (0002) diffraction peak at 26.7° in XRD analysis, accompanied by AlN signatures generated during sapphire nitridation pretreatment, whose buffer layer serves to alleviate lattice mismatch with the substrate. The UV–visible transmission spectrum presented in Figure 1f reveals a distinct knee point at approximately 215 nm, followed by a rapid transmission decrease, characterizing the optical behavior of h‐BN. The calculated bandgap energy from the Tauc plot methodology is 5.76 eV, aligning with literature values for multilayer h‐BN structures [27].

**FIGURE 1:**
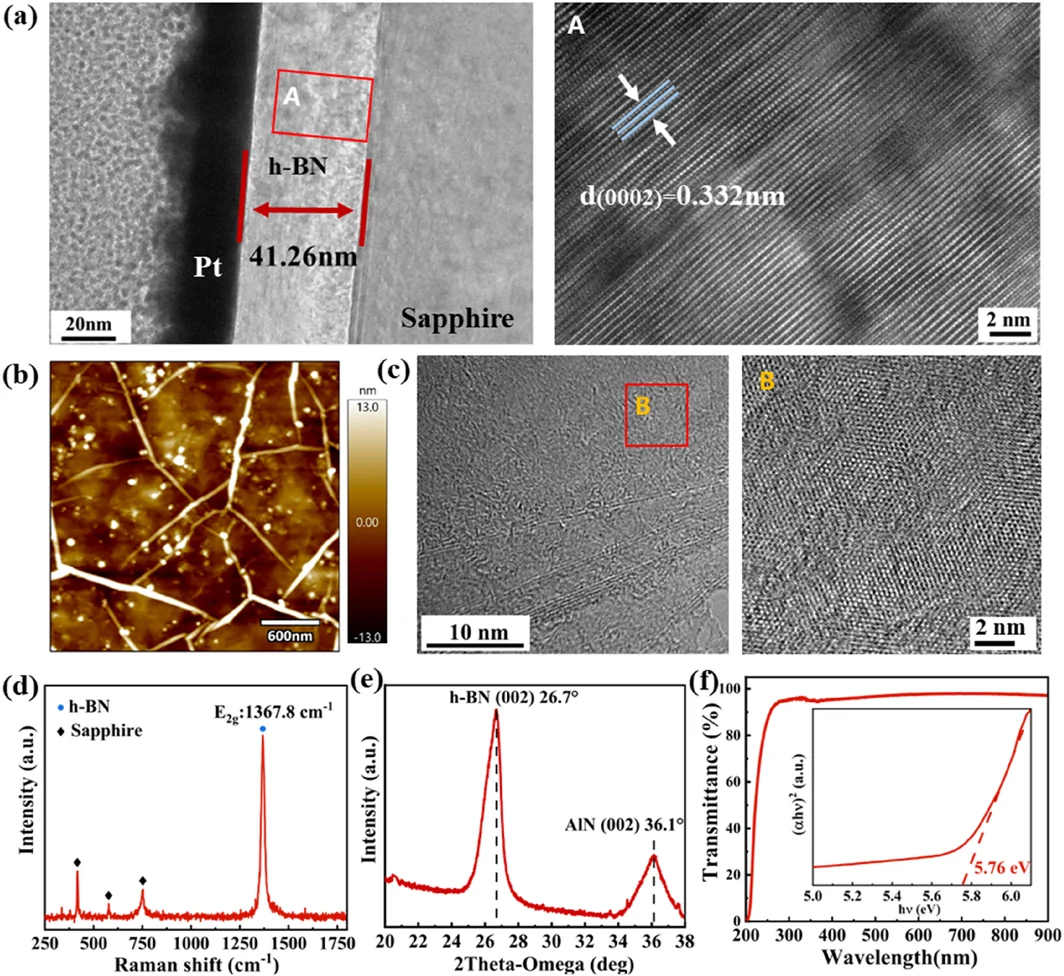
Characterization of h‐BN films grown by MOCVD: (a) cross‐sectional TEM image of the h‐BN film; (b) AFM image showing the surface morphology; (c) the in‐plane TEM image of ultrasonically exfoliated h‐BN; (d) Raman spectrum; (e) XRD of the h‐BN film; (f) transmission spectrum.

XPS analysis was performed on the h‐BN thin film, prior to and after the fabrication of Pt nanoparticles by thermal dewetting, to examine the effect of the Pt nanoparticles on the surface defects of h‐BN. As depicted in Figure 2, the primary spectral features are observed at binding energies of approximately 190.5 eV (B‐N), 398.0 eV (N‐B), 531.5 eV (O‐B), and 532.9 eV (O‐C). The surface states of the h‐BN were modified by the generation of platinum nanoparticles in the thermal dewetting process. Diverse point defects (e.g., vacancies, antisite defects, and substitutional impurities) were generated by the formation of Pt nanoparticles. This is evidenced by minor shifts (∼0.1 eV) observed in the B 1s and N 1s binding energies; the B‐N bond binding energy decreased from 190.5 to 190.4 eV (Figure 2a), whereas the N‐B bond binding energy shifted from 398.1 to 398.0 eV (Figure 2b). Additionally, a notable broadening of the full width at half maximum (FWHM) for the B‐N‐ and N‐B‐related peaks was observed. The FWHM for the B‐N bond broadened from 1.11 to 1.19 eV and for the N‐B bond from 1.11 to 1.14 eV, as illustrated in Figure 2a,b. Specifically, some defects within the h‐BN film are manifested as the substitution of nitrogen lattice sites by oxygen atoms (forming O_B_ defects) or the combination of oxygen atoms with dangling bonds from boron at defect locations. Direct evidence supporting this is provided by the observed increase in O‐B bond content, which is presented in Figure 2c. At the same time, the surface reactivity of the h‐BN was enhanced. The defect sites served as highly reactive centers, by which ambient carbon/oxygen‐containing species were adsorbed. This is evidenced by an increase in the O‐C bond content, as shown in Figure 2c.

**FIGURE 2:**
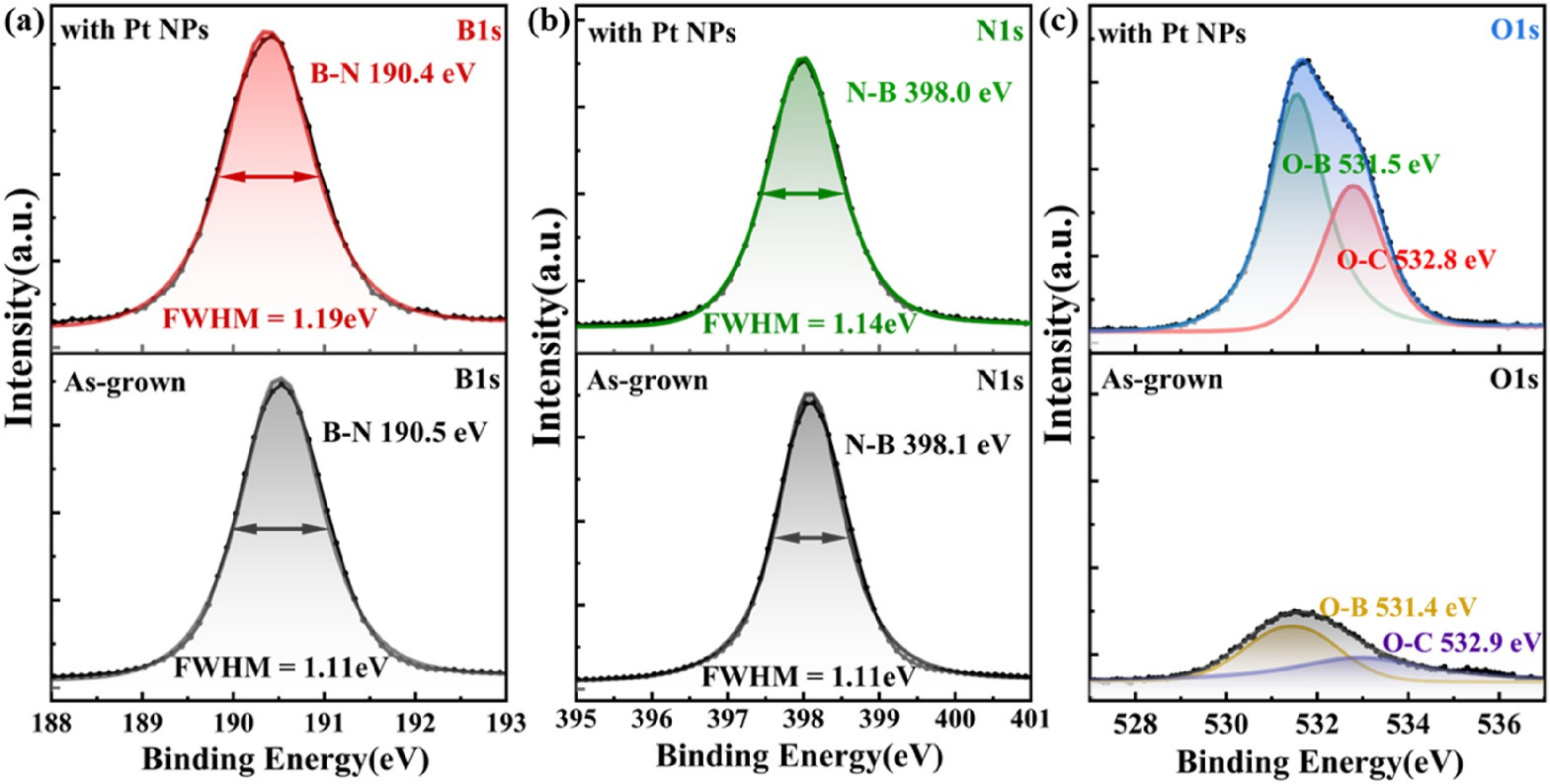
XPS spectra of the h‐BN thin film acquired before and after the formation of Pt nanoparticles via the thermal dewetting process: (a) B 1s; (b) N 1s; (c) O 1s.

Pt films with three thicknesses (approximately 2, 3, and 4 nm) were deposited on h‐BN thin films, and the AFM surface morphologies of Pt nanoparticles (NPs) after annealing are shown in Figure 3a–c. The h‐BN thin films exhibit smooth surfaces where Pt NPs with quasi‐circular shapes are randomly distributed in size and position. The Pt NP grain size progressively increases with original Pt film thickness during the dewetting process. The corresponding particle size histograms in Figure 3d–f statistically demonstrate that the mean diameters (based on normal distribution fitting) of Pt NPs derived from ∼2, 3, and 4 nm Pt films through the dewetting process are 32 nm, 50 nm, and 85 nm, respectively. In which the horizontal axis denotes the particle size in nanometers, and the vertical axis represents frequency, calculated as the percentage derived from the ratio of the count of Pt NPs of a specific size to the total count of all Pt NPs, multiplied by 100%. VUV PD devices fabricated with these characteristic dimensions are designated as D32, D50, and D85.

**FIGURE 3:**
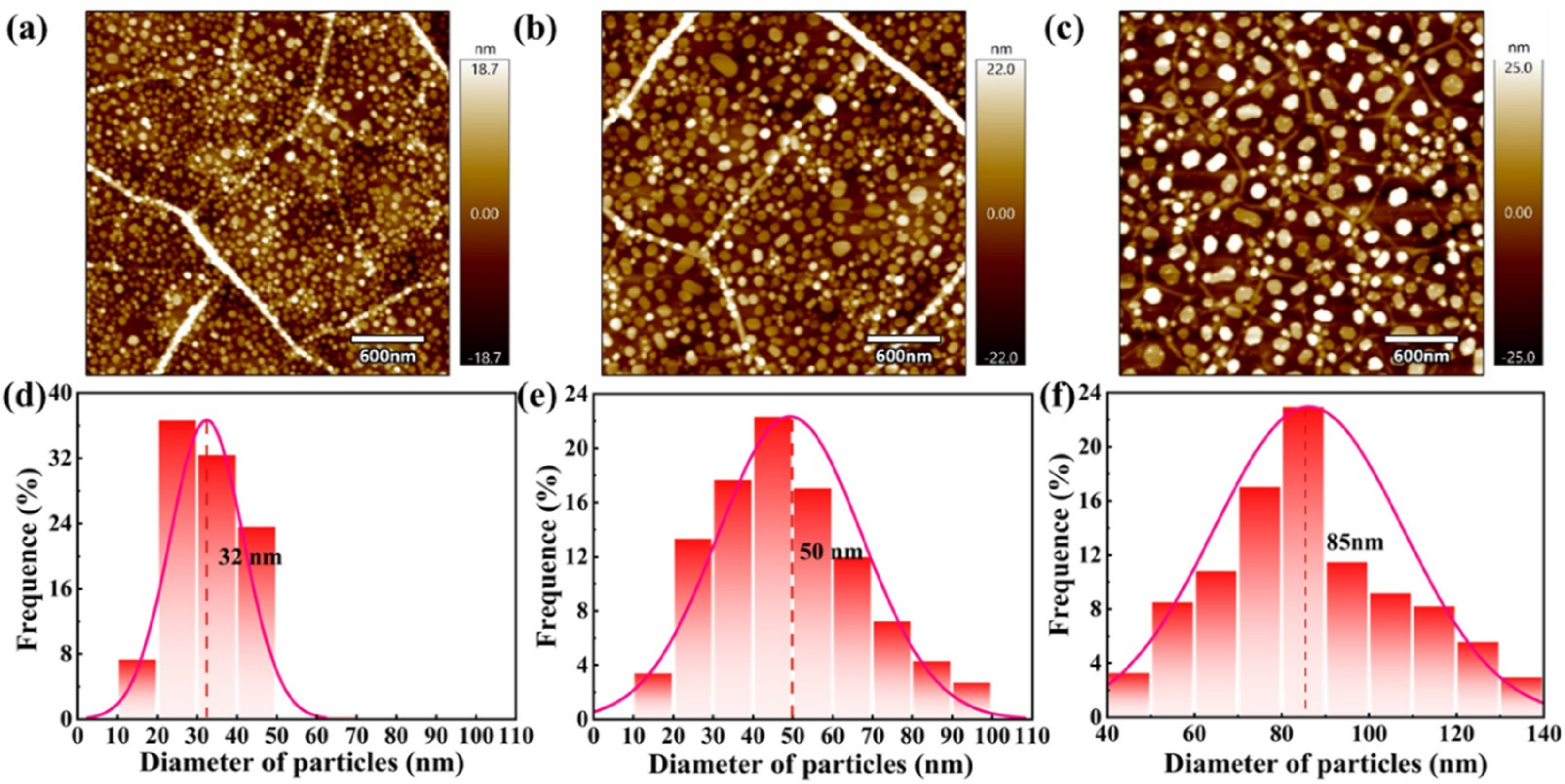
Panels (a) and (d), (b) and (e), and (c) and (f) present the AFM images of surface morphology and the corresponding particle size distributions of Pt NPs after annealing of 2 nm‐, 3 nm‐, and 4 nm‐thick Pt films, respectively.

To construct h‐BN‐based PDs with/without NPs, gold electrodes (40 nm thickness) arranged in MSM geometry with 30 μm interdigitated spacing were deposited on both pristine and NP‐modified film surfaces, as schematically shown in Figure 4a. Electrical characterization was performed for all four PD configurations under both dark conditions and 185 nm VUV light irradiation. The voltage‐dependent photocurrent (*I*_ph_) responses at 185 nm illumination are presented in Figure 4b. A remarkable *I*_ph_ enhancement under high bias voltages was clearly observed in NP‐incorporated devices, attributable to the Pt NPs’ presence. The NP‐mediated dark current (*I*_dark_) characteristics are displayed in Figure 4c. MSM PDs fabricated on D32, D50, and D85 substrates showed *I*_dark_ values of 0.10 pA, 0.25 pA, and 6.69 pA, respectively, whereas the reference h‐BN PD without NPs exhibited an ultralow *I*_dark_ of 0.03 pA. A systematic dark current elevation was detected in NP‐containing devices. Notably, PDs constructed on D85 substrates demonstrated significantly increased dark current levels. This increased dark current likely originates from defect states introduced during NP decoration on the h‐BN matrix. The interface between Pt NPs and h‐BN may create defect states that facilitate charge carrier trapping and introduce additional current transport mechanisms, thereby increasing dark current. Figure 4d demonstrates that under 185 nm irradiation, the photocurrent exhibits substantial enhancement with increasing power density, whereas the dark current maintains consistently low values, indicating a direct proportionality between photogenerated carriers and photon absorption. Through power‐law fitting of the *I*_ph_∝P^*α*^ relationships, the extracted exponents *α* for MSM photodetectors fabricated on D32, D50, and D85 substrates were determined as 0.98, 0.99, and 1.00, respectively. For MSM devices fabricated from as‐grown h‐BN films, the derived power‐law exponent *α* reached 1.06. The proximity of *α* values to 1 indicates minimal photocurrent attenuation caused by defect‐mediated carrier recombination within the films. Beyond current‐voltage measurements, we systematically characterized the temporal current response (*I*–*t* curves) of the photodetectors under fixed bias conditions. The temporal response measurements were conducted by periodically modulating the 185‐nm vacuum ultraviolet (VUV) light source (without mechanical chopping) and synchronously recording the photocurrent transients. Figure 4e presents the photodetectors' dynamic response to modulated optical stimuli. The plot demonstrates reliable and effective responses of the PDs to the modulated optical input, highlighting their operational stability. At 20 V applied bias with 0.67 mW cm^−2^ illumination, Pt‐functionalized MSM PDs on D32, D50, and D85 substrates exhibited photocurrents of 1.48, 1.04, and 0.77 nA, respectively, revealing 111%, 49%, and 10% enhancement compared to the 0.70‐nA baseline current from the pristine h‐BN device, demonstrating the efficacy of Pt nanoparticle modification. After the observation of stable temporal responses, we calculated the rise and fall times for each PD. As the measured response times were limited by the light source switching speed, the actual PD response is expected to be faster than the calculated values. The rise time *τ*_r_ (decay time *τ*_d_) is defined as the time interval required for the detector output to rise from 10% (fall from 90%) to 90% (10%) of its maximum value. Using this criterion, *τ*_r_ and *τ*_d_ for the pristine h‐BN‐based MSM PD were calculated as 215.50 and 41.29 ms, respectively, as shown in Figure 4f. For Pt NP‐modified devices, *τ*_r_ values of 214.15, 210.5, and 207.43 ms with corresponding *τ*_d_ values of 21.45, 41.78, and 41.46 ms were obtained for D32‐, D50‐, and D85‐based MSM PDs, respectively (Figure 4g–i). This allows us to conclude that the detector speed remains unaffected by Pt NP surface modification on h‐BN, showing comparable performance to PDs fabricated on pristine h‐BN.

**FIGURE 4:**
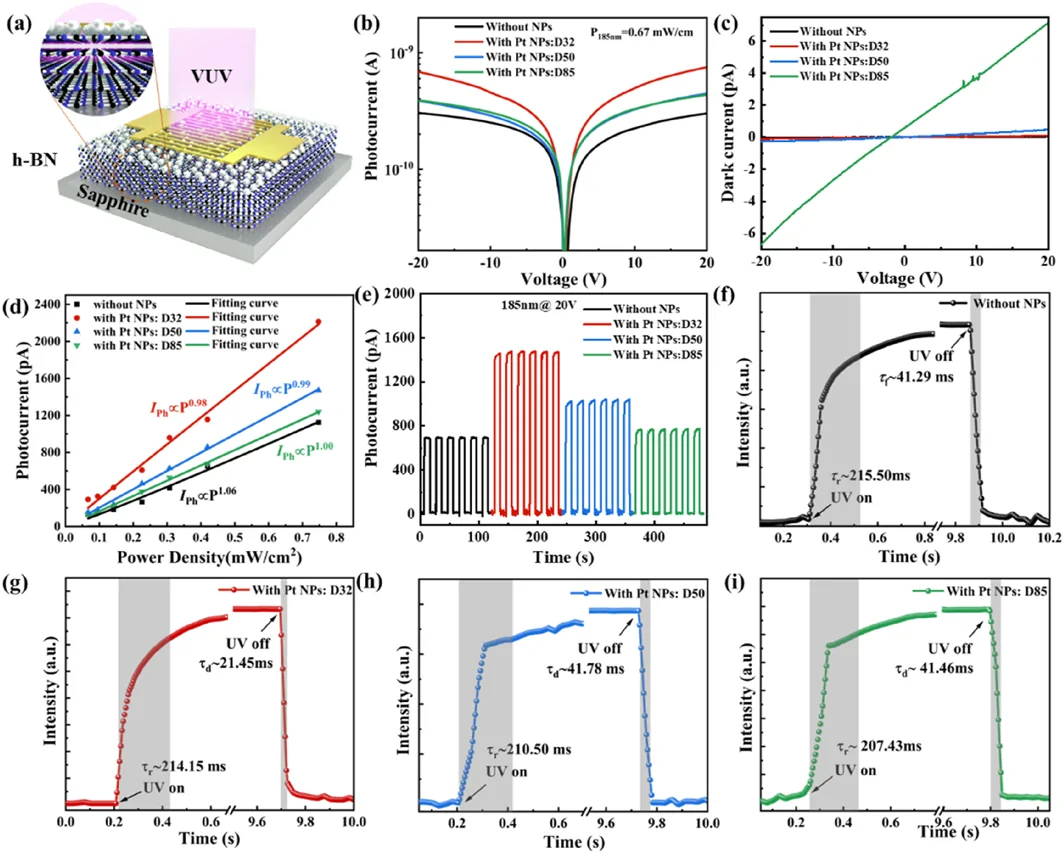
(a) Schematic diagram of the VUV MSM PD; (b) *I*–*V* characteristics of MSM PDs fabricated on pristine h‐BN (without NPs) and modified h‐BN with Pt NPs (D32, D50, and D85) under 185 nm illumination; (c) dark *I–V* characteristics of the four PDs; (d) power‐law dependence of photocurrent (*I*_ph_) on incident optical power density for the MSM PD fabricated on h‐BN thin films with and without Pt NPs; (e) temporal response characteristics under 185 nm VUV irradiation (0.67 mW cm^−2^) at 20 V bias, illustrating the rise and decay times of the MSM PD photocurrent; time‐resolved response (f) device without Pt NPs, (g) with Pt NPs (D32), (h) with Pt NPs (D50), and (i) with Pt NPs (D85).

The photodetection performance was quantitatively evaluated through derivation of responsivity (*R*) and specific detectivity (D*) [35, 36, 37]. The parameters R and D* are expressed as follows:

(1) $$R=\frac{I_{\text{ph}}-I_{\text{dark}}}{P_{\lambda }},$$

(2) $$D^{*}=\frac{\sqrt{A}R}{\sqrt{{2eI}_{\text{dark}}}},$$

where *I*_ph_ and *I*_dark_ correspond to photocurrent and dark current, respectively. *P*_*λ*_ represents illumination intensity, *λ* is incident wavelength, and e denotes the elementary charge. *A* is the PD's effective area (1.3 × 10^−3^ cm^2^). Figure 5a reveals null photoresponse in the 254–355‐nm spectral range, whereas the responsivity progressively increases as the wavelength shortens from 254 to 185 nm (vacuum ultraviolet region). At 185 nm illumination, the Pt‐functionalized detectors demonstrated peak responsivities of 1.02 mA/W (D32), 0.56 mA/W (D50), and 0.47 mA/W (D85), significantly exceeding the 0.42‐mA/W baseline value from the unmodified h‐BN device under equivalent measurement conditions. Remarkably, this translates to 143% peak responsivity enhancement through Pt nanoparticle integration, verifying their plasmonic enhancement effects. As evidenced in Figure 5b, the Pt‐modified D32 detector achieved a detectivity (D*) of 2.95 × 10^11^ Jones at 185 nm, demonstrating 117% improvement over the baseline h‐BN device (1.36 × 10^11^ Jones), highlighting interfacial engineering benefits. In contrast, D85‐modified devices suffered 86% detectivity degradation (D* = 1.83 × 10^10^ Jones), indicating substrate‐dependent optimization requirements. Equation (2) indicates that at fixed wavelength, detectivity scales proportionally with responsivity and inversely with the square root of dark current. Although NPs on D85 induced greater responsivity, defect states caused a substantial dark current increase, ultimately reducing D*. Performance comparisons with large‐area h‐BN‐based PDs and other VUV detectors in response time and detectivity are shown in the inset of Figure 5c, demonstrating our PD's excellence.

**FIGURE 5:**
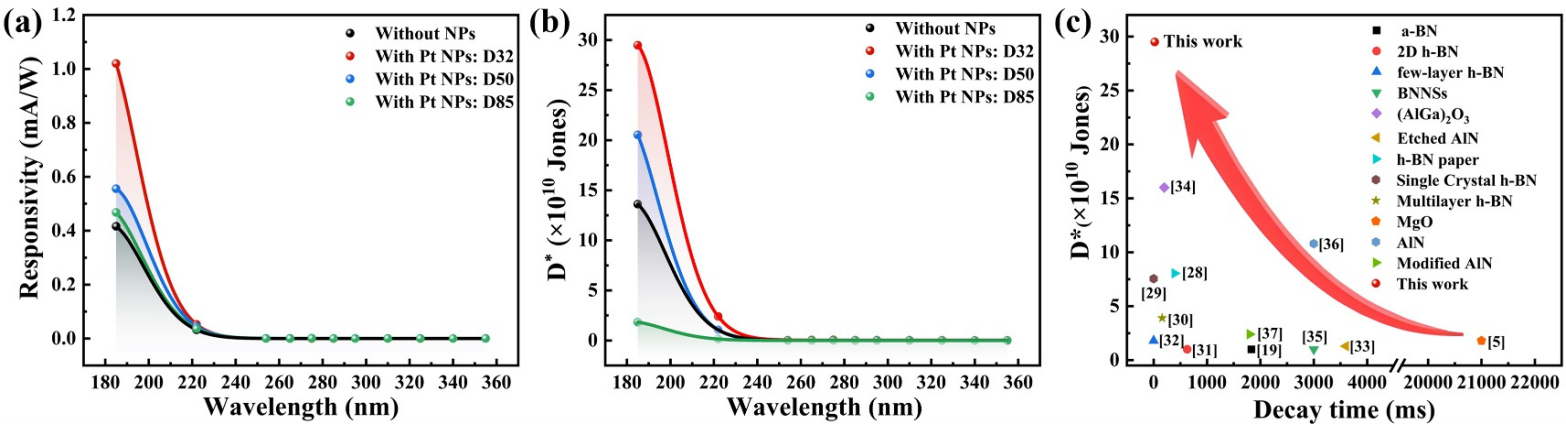
(a) and (b) plots of responsivity and specific detectivity (D*) as functions of incident wavelength in the UV region (185–360 nm), measured at a bias voltage of 20 V; (c) The performance comparation of large‐area h‐BN PDs and other VUV PDs in terms detectiviy and response speed, and the arrow points in the direction of high‐performance. Besides the aforementioned devices, photodetectors (PDs) based on large‐area hexagonal boron nitride (h‐BN) and related materials are also summarized. These include: amorphous h‐BN deposited via magnetron sputtering [19]; single‐crystal h‐BN grown by submicron‐spacing vapor deposition [28]; h‐BN paper fabricated via mechanical pressing [24]; 2D h‐BN synthesized by ion beam sputtering deposition [29]; few‐layer h‐BN produced via ion beam sputtering deposition [30]; multilayer h‐BN films prepared by ambient pressure chemical vapor deposition [31]; boron nitride nanosheets (BNNSs) obtained via digitally controlled pulsed laser plasma deposition [32]; 2D MgO formed through conformal anneal synthesis [5]; (AlGa)_2_O_3_/Ga_2_O_3_ heterostructures grown by MOCVD [25]; etched AlN fabricated via flow rate modulation epitaxy [26]; as well as AlN [33] and modified AlN [33] layers deposited by MOCVD.

Theoretical analysis was conducted to investigate the plasmonic enhancement mechanism arising from platinum nanoparticle‐mediated LSPR in h‐BN MSM photodetectors. Accordingly, Figure 6a,b, respectively, presents the calculated extinction data, cross‐sectional profiles, and interfacial electric field distributions for D85‐, D50‐, and D32‐correlated Pt NPs under 185 nm illumination. Clear observations reveal strong LSPR coupling of Pt NPs in the VUV regime, with smaller D32 particles exhibiting enhanced absorption coupling and larger extinction cross‐sections compared to the D50 and D85 counterparts. Regarding field distributions, the near‐field enhancement from Pt‐LSPR coupling progressively intensifies from D85 to D32 configurations, consistent with their extinction characteristics. Crucially, it has been demonstrated that incident VUV photons can be coupled by Pt NPs via electromagnetic wave penetration through NP cross‐sections, significantly enhancing h‐BN film absorption.

**FIGURE 6:**
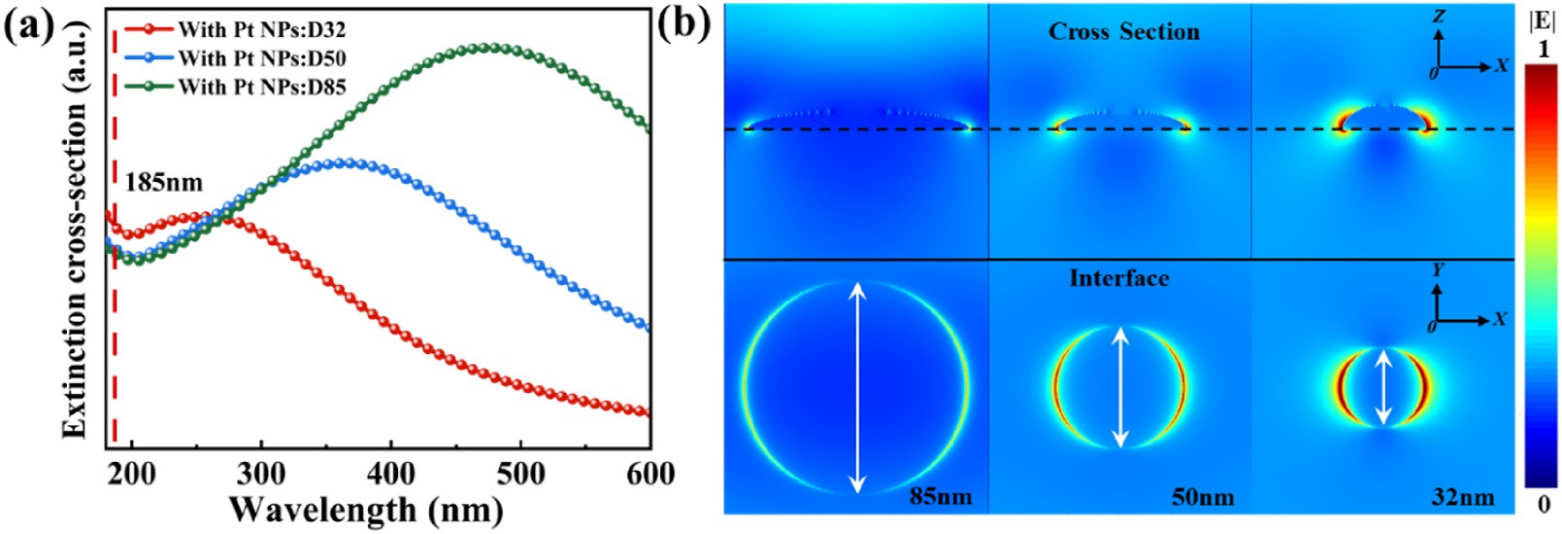
(a) Simulated spectral extinction profiles of Pt nanoparticles (NPs) with different diameters; (b) simulated electric field distributions of the *x*–*z* and *x*–*y* planes for Pt nanoparticles (NPs) with varying diameters.

## 3 Conclusion

In summary, we have successfully demonstrated for the first time a plasmon‐enhanced VUV PD based on h‐BN films utilizing LSPR in Pt nanoparticles. The nanoparticles were synthesized via a cost‐effective and efficient thin‐film dewetting method, modifying MOCVD‐grown h‐BN through controlled evaporation of Pt films with varying thicknesses. NPs dewetted from 2, 3, and 4 nm films showed average diameters of 32, 50, and 85 nm, respectively. VUV PDs based on pristine h‐BN and modified versions (D32/D50/D85 with 32/50/85 nm NPs) underwent comprehensive optoelectronic characterization. At 20 V bias with 0.67 mW/cm^2^ illumination, D32 devices exhibited 1.02 mA/W responsivity (143% enhancement) and 2.95 × 10^11^ Jones detectivity (117% improvement). Numerical simulations of extinction characteristics and electric field distributions at 185 nm VUV revealed the enhancement mechanism: LSPR in NPs improves light absorption and local field intensity, boosting photocurrent generation. Furthermore, even with Pt‐modified h‐BN surfaces, we believe plasmonic enhancement will play vital roles in addressing the low responsivity of 2DLM‐based VUV devices, and our study represents a significant step in this direction.

## 4 Experimental Section

*Growth of* *h‐BN films by MOCVD and subsequent thermal annealing*: Approximately 40 nm of h‐BN were grown on 2‐inch double‐side‐polished sapphire substrates by using a commercial MOCVD system at 1215°C under a reactor pressure of 70 mbar with H_2_ carrier gas. A pulsed source‐injection mode composed of two steps in a single pulse cycle was employed: (1) injection of 20 sccm triethylborane (TEB) for 2 s, followed by (2) injection of 2000 sccm NH_3_ for 4 s. A total of 1200 pulse cycles resulted in the growth of h‐BN. The as‐grown h‐BN film, deposited via MOCVD, was subjected to thermal annealing at 1650°C for 120 min in an argon (Ar) atmosphere at atmospheric pressure, with an Ar gas flow rate of 200 sccm, as shown in Supporting Information S1: Figure S1a.

*Pt nanoparticles (NPs) of varying sizes were prepared on h‐BN films:* Pt films with nominal thicknesses of approximately 2, 3, and 4 nm were deposited on the h‐BN surface using electron beam evaporation with a base pressure of 10^−6^ Torr. Subsequently, the samples were subjected to rapid thermal annealing at an optimized temperature of 850°C for 300 s in a nitrogen atmosphere. This process induced dewetting of the Pt film, resulting in the formation of Pt NPs on the h‐BN films, as shown in Supporting Information S1: Figure S1b,c.

*Fabrication of VUV MSM photodetectors (PDs):* A 40‐nm Au metal layer was evaporated onto both pristine and Pt NP‐modified samples through a mask to define an interdigitated electrode geometry characteristic of metal–semiconductor–metal (MSM) structures, with an electrode width and interelectrode spacing of 30 μm, as shown in Supporting Information S1: Figure S1d.

*Characterization*: The surface morphology of the h‐BN films was characterized using a Jupiter XR atomic force microscope (AFM), and the microstructure of the h‐BN/sapphire heterostructures was examined using a Tecnai G2 F30 transmission electron microscope (TEM). Raman spectroscopy was performed using a confocal Raman microscope (alpha300R, WITec) with a 532‐nm laser as the excitation source. Absorption spectra were recorded using a UV–Vis spectrophotometer (Lambda 35, PerkinElmer). X‐ray diffraction (XRD) measurements were conducted using a SmartLab 9 kW diffractometer (Rigaku) to confirm the composition of the Pt NP/h‐BN/sapphire structure. Photodetection measurements were conducted under illumination from a low‐pressure mercury lamp emitting at 185 nm, whereas spectral selectivity was evaluated using a 222‐nm KrCl excimer lamp and deep‐UV LEDs emitting in the range of 254–360 nm. The optical power density of the illumination sources was measured using a calibrated optical power meter (Thorlabs PM100 USB, S401C, and ES120C). Electrical and optoelectronic measurements (*I–t* and *I–V* curves) were obtained using a Keithley 2902 and a Keithley 4200 source meter, respectively. Simulations were conducted via the 3D finite‐difference time‐domain method implemented in Ansys Lumerical 2025 R1, wherein the refractive indices of materials were sourced from published references. The diameters of the hemispherical nanoparticles were designed with three size gradients: 32, 50, and 85 nm. The computational region was configured to fully cover the structural model, whereas boundary conditions were implemented using perfectly matched layers (PML). Electromagnetic simulations captured both the electric field profiles and extinction spectra of platinum nanoparticles under periodic arrangement across varying wavelengths.

## Author Contributions

**Zhiwei Gao:** writing – original draft, conceptualization, methodology, data curation, investigation, validation, formal analysis. **Yufan Wei:** resources, formal analysis. **Weijie Liu:** resources. **Yongming Zhao:** resources. **Zhengang Liang:** resources. **Yuhui Zeng:** resources. **Zhencheng Li:** resources. **Yang Peng:** resources. **Feng Wu**: writing – review and editing. **Hao‐Chung Kuo**: writing – review and editing. **Jiangnan Dai**: conceptualization, methodology, funding acquisition, resources.

## Funding

This work was financially supported by the National Natural Science Foundation of China (Grant No. 62574090).

## Supporting information


Supporting Information S1

## Data Availability

The data that support the findings of this study are available from the corresponding author upon reasonable request.
