# Supplementary material for: High‐Detectivity MOCVD‐Epitaxial h‐BN‐Based Vacuum UV Photodetectors With Pt Nanoparticle‐Engineered Localized Surface Plasmon Resonance Enhancement
Source: Nanophotonics. 2026 Apr 14;15(8):e70070. doi: 10.1002/nap2.70070 (PMC13642482; doi:10.1002/nap2.70070)
Supplement: Supplementary file 1 [file NAP2-15-e70070-s001.docx]

Supporting Information

High-Detectivity MOCVD-Epitaxial h-BN-Based Vacuum UV Photodetectors with Pt Nanoparticle-Engineered Localized Surface Plasmon Resonance Enhancement

*Zhiwei Gao^1^, Yufan Wei^1^, Weijie Liu^1^, Yongming Zhao^1^, Zhengang Liang^1^, Yuhui Zeng^1^, Zhencheng Li^1^, Yang Peng^2^, Feng Wu^1,*^, Hao-Chung Kuo^3,*^ and Jiangnan Dai^1,*^*

[1] Z. Gao, Y. Wei, W. Liu, Y. Zhao, Z. Liang, Y. Zeng, Z. Li, Prof. F. Wu, Prof. J. Dai

Wuhan National Laboratory for Optoelectronics, Huazhong University of Science and Technology, Wuhan, 430074, China.

E-mail: [wufeng123@hust.edu.cn](mailto:wufeng123@hust.edu.cn);

daijiangnan@hust.edu.cn (J. Dai);

[2] Prof. Y. Peng

School of Aerospace Engineering, Huazhong University of Science and Technology, Wuhan 430074, China.

[3] Prof. H.Guo

Department of Photonics, Institute of Electro-Optical Engineering, National Yang Ming Chiao Tung University, Hsinchu 30010, Taiwan

E-mail: hckuo0206@nycu.edu.tw

**Figure S1**. Schematic illustration of the preparation process for the MSM photodetector (PD) incorporating Pt NPs: **(a)** deposition of h-BN on sapphire; **(b)** deposition of a Pt layer on h-BN/sapphire; **(c)** formation of Pt NPs on h-BN/sapphire; **(d)** fabrication of the MSM photodetector with Pt NPs.

The thermally activated dewetting behavior of 2 nm Pt films is systematically investigated in Figure S2 through SEM characterization after 60 seconds annealing at temperature gradients spanning 800-950°C. The critical temperature for Pt dewetting into nanoparticles is identified as 850°C (Figure S2(b)), below which the film remains continuous (Figure S2(a)), while higher temperatures show negligible impact on NP morphology (Figure S2(c,d)), establishing 850°C as the optimal annealing temperature. Figure S3 presents the SEM images of 2 nm Pt films annealed at 850°C for 30 s, 60 s, 300 s, and 600 s. Incomplete dewetting occurs after 30 s (Figure S3(a)), while 60 s annealing initiates NP formation with residual elongated structures (Figure S3(b)). Complete dewetting into discrete NPs is achieved at 300 s (Figure S3(c)), with no significant morphological changes observed at 600 s (Figure S3(d)), thus identifying 300 s as the optimal duration. Figure S4 demonstrates thickness-dependent NP size control through 850°C/300-s annealing of Pt films with varying initial thicknesses (1-5 nm). The observed thickness-proportional NP scaling provides a robust platform for graded plasmonic nanostructure engineering. As shown in Figure S5, XRD 2θ−ω scans of h-BN films with/without Pt NPs reveal that the Pt (39.6°) diffraction peak intensity enhances with increasing NP size, while the h-BN (26.5°) and sapphire (41.6°) peaks remain stable, confirming well-controlled experimental conditions for investigating Pt NP effects on subsequent VUV MSM photodetector performance. Shown in Figure S6 are the XPS survey scan analysis results for the samples corresponding to those in Figure 2. A significant change in the elemental composition of the h-BN film surface was induced following the deposition of Pt nanoparticles. Specifically, an increase in nitrogen content and a decrease in oxygen content were observed, which suggests that nitrogen sites within the h-BN lattice were replaced by oxygen atoms, leading to the formation of O_B_ defects. At the same time, a notable rise in carbon content was detected, indicating enhanced surface reactivity of the h-BN. The defect sites, serving as highly reactive centers, facilitated the adsorption of ambient carbon/oxygen-containing species. Presented in Figure S7 are the extinction coefficient curves derived from FDTD simulations, illustrating the effect of varying nanoparticle density on the optical characteristics for a fixed Pt nanoparticle diameter of 32 nm. Within the vacuum ultraviolet (VUV) range of 180–200 nm, the extinction coefficient was found to decrease as the wavelength increased. In the 200–600 nm region, the extinction coefficient initially increased and subsequently decreased, with a peak value being attained at 275 nm. At the specific wavelength of 185 nm, the extinction coefficient was observed to first increase and then decrease with decreasing Pt nanoparticle density (corresponding to an increasing inter-particle gap), and the optimal value was obtained when the inter-particle distance was 92 nm. Further investigation into the extinction properties of Pt nanoparticles with various diameters, while maintaining a constant inter-particle distance, was conducted using FDTD simulations, the results of which are displayed in Figure S8. The simulation encompassed nanoparticle diameters from 10 to 85 nm. Under the premise of a periodic arrangement of particles and an ideal interface, the Pt nanoparticles with a size of 32 nm were found to demonstrate the most pronounced plasmonic resonance enhancement within the VUV band, which was manifested as the maximum extinction coefficient. Of particular note, this trend was not observed to continue infinitely as the particle size was decreased. For particle sizes below approximately 20 nm, a substantial weakening and broadening of the plasmonic resonance was induced by increased surface scattering and quantum confinement effects. This observed trend was corroborated by the simulation data, which showed that the enhancement effect for the 10 nm and 20 nm particles was inferior to that of the 32 nm particles. Simulated in Figure S9 is the change in the extinction coefficient resulting from the coupling of Pt nanoparticles with varying diameters to the h-BN thin film. A comparison with Figure S8 reveals that the extinction characteristics are dominated by the h-BN film, and the overall extinction coefficient exhibits a gradual decrease with increasing wavelength. At the wavelength of 185 nm, a distinct enhancement of the extinction coefficient was achieved by the incorporation of Pt nanoparticles. The most substantial enhancement was observed for the 32 nm particles, a finding that is in agreement with the results presented in Figure 6(a). Presented in Figure S10 are the quantum efficiency curves for h-BN VUV MSM photodetectors modified with Pt nanoparticles of various diameters. These curves were derived from calculations using the data in Figure 5(a) and the following formula:

$$\eta=\frac{R\cdot hc}{q\lambda}\times100\%$$

Where η represents the quantum efficiency, R is the responsivity, λ denotes the wavelength of the incident light, h is Planck's constant (6.626×10⁻³⁴ J·s), c is the speed of light (3×10⁸ m/s), and q is the elementary charge (1.602×10⁻¹⁹ C). It is illustrated in Figure S10 that the quantum efficiency of the VUV photodetectors was significantly enhanced by the plasmonic effect of the Pt nanoparticles. A maximum quantum efficiency of 0.68% was achieved for the Pt NPs@ h-BN device with a nanoparticle diameter of 32 nm. This value corresponds to a 143% enhancement in quantum efficiency relative to the pristine h-BN device that was not modified with Pt NPs.

**Figure S2.** The SEM surface morphology of Pt NPs obtained under different annealing temperatures: **(a)** 800℃, **(b)** 850℃, **(c)** 900℃ and **(d)** 950℃.

**Figure S3.** The SEM surface morphology of Pt NPs obtained under different annealing times: **(a)** 30s, **(b)** 60s, **(c)** 300s, **(d)** 600s

**Figure S4.** The SEM surface morphology of Pt NPs obtained after annealing of different Pt film thicknesses: **(a)** 0 nm, **(b)** 2 nm, **(c)** 3 nm, and **(d)** 4 nm

**Figure S5**. X-ray diffraction (XRD) patterns of Pt NPs@h-BN/sapphire with different particle sizes.

**Figure S6.** The full spectra of XRD Figure 2

**Figure S7.** Simulated extinction spectra of Pt nanoparticles with different interparticle spacings.

**Figure S8.** Simulated spectral extinction profiles of Pt nanoparticles (NPs) with different diameters

**Figure S9.** Simulated extinction spectra of Pt nanoparticles coupled with an h-BN film for various nanoparticle diameters.

**Figure S10.** Quantum efficiency curves of Pt NPs@h-BN VUV MSM photodetectors with Pt nanoparticles of different diameters
